# Foundation and Multimodal Models for Drug Discovery in Molecular Informatics: Principles, Evaluation, and Practical Guidance

**DOI:** 10.1002/minf.70027

**Published:** 2026-03-25

**Authors:** Emmanuel Pio Pastore, Francesco De Rango

**Affiliations:** ^1^ University of Calabria Department of Biology Ecology and Earth Science Rende Italy

**Keywords:** benchmarking, chemical language models, diffusion models, drug discovery, foundation models, multimodal learning

## Abstract

Foundation and multimodal models are rapidly becoming a core methodology in molecular informatics, particularly for drug discovery, by leveraging large‐scale pretraining across sequences, graphs, 3D structures, and text. This mini‐review provides practical guidance on when these models help, how to choose representations and data, and how to design pretraining and adaptation pipelines for real‐world use. We clarify what qualifies as a foundation model in chemistry; compare chemical language models, graph‐based architectures, and 3D equivariant networks; review multimodal strategies that connect molecules with proteins, pockets, and natural language; and summarize diffusion‐based generative modeling. We also emphasize rigorous evaluation, discussing realistic splitting protocols, distribution shift, activity cliffs, uncertainty calibration, and conformal prediction in the context of widely used benchmarks.

## Graphical abstract

The graphical abstract summarizes how foundation models pretrained on large molecular data provide transferable representations. Through multimodal learning, these representations integrate molecular structure, biological context, and text‐derived knowledge, enabling downstream drug discovery tasks such as property prediction, target interaction modeling, and molecular design.

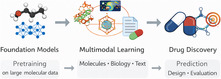

## 1 Introduction

In molecular informatics, representation and data have long dictated what models are able to learn and how reliably they can generalize. Over the past decade, progress has often been driven less by architectural innovations than by the quality, scale, and consistency of the underlying molecular corpora. The recent emergence of “foundation models", systems pretrained on broad, heterogeneous chemical and biological data and subsequently adapted to many downstream tasks, has begun to shift this balance by enabling strong cross‐domain generalization from a single backbone trained once at scale [1, 2]. These models integrate principles from natural‐language processing, geometry‐aware learning, and protein language modeling, creating a unified computational space in which molecules, proteins, and text‐based annotations can be treated as mutually informative modalities rather than isolated silos.

For chemistry and biology, the opportunity is to connect molecular strings, graphs, and three‐dimensional conformers with protein sequences, pocket structures, reaction contexts, and free‐text descriptions within a coherent pretraining and evaluation framework. Such integration allows models to exploit relationships that are implicit in experimental data, chemical series evolution, target families, shared assay formats, and protocol‐level cues, while preserving the physical constraints that govern molecular behavior. At the same time, it raises new challenges around data standardization, provenance, leakage, and the interpretability of high‐capacity architectures. Recent reviews have discussed molecular representations and their implications for generalization in molecular machine learning [3]. This mini‐review concentrates on foundation and multimodal pretraining for drug discovery, with emphasis on evaluation under realistic splits and on reporting choices that enable fair comparisons.

## 2 Molecular and Biological Representations That Scale and Benchmarking

Choice of representation fundamentally constrains what a model is able to learn, how it encodes chemical knowledge, and the types of generalization it can express. Line notations such as SMILES (Simplified Molecular Input Line Entry System) remain ubiquitous thanks to their compact syntax, direct compatibility with cheminformatics tooling, and ease of storage and exchange across databases and workflows [4]. However, the sensitivity of SMILES to tokenization choices and the presence of multiple syntactic forms for the same molecule can introduce instabilities during pretraining or generation. This motivated the development of robust alternatives such as SELFIES, which provide a guaranteed‐valid molecular string representation and reduce the need for post‐hoc validity checks during decoding [5]. These representations also simplify augmentation strategies, randomization, canonical/non‐canonical variants, and stereochemical perturbations, which in turn support more diverse and chemically consistent training corpora.

Graph‐based encodings complement string approaches by operating directly on atom‐ and bond‐level structures, typically constructed with established cheminformatics toolkits such as RDKit [6]. Graph representations expose fine‐grained connectivity, hybridization states, ring systems, and local physicochemical features that are difficult to express cleanly in sequence form. Because they are easy to canonicalize and featurize, graph encodings lend themselves well to dataset‐wide curation pipelines. These same pipelines underpin curated bioactivity resources such as ChEMBL, which provide broad assay coverage, explicit provenance, and standardized target annotations essential for pretraining and evaluation across multiple biological contexts [7].

Neural architectures have evolved alongside these representations. Message‐passing networks offer a strong and widely validated baseline for small‐molecule property prediction [8], while continuous‐filter and directional message‐passing architectures better capture geometry‐aware interactions and directional dependencies central to quantum‐chemical and binding‐site modeling [9, 10]. More recently, 3D‐equivariant neural networks have enabled direct learning on three‐dimensional structures, enforcing rotational and translational symmetries that mirror the underlying physics of molecular systems. These architectures improve pose awareness, conformational reasoning, and binding‐site sensitivity without explicitly hand‐crafting invariant features.

Reliable benchmarking is equally dependent on representation. MoleculeNet, the OGB molecule suites, and ligand‐based design frameworks provide diverse tasks spanning quantum properties, ADMET (absorption, distribution, metabolism, excretion, toxicity) endpoints, toxicology, and bioactivity classification, accompanied by principled data splits that go beyond random partitioning [11, 12]. Such splits, scaffolds, chronologies, and family‐awareness, better reflect the distribution shifts encountered in medicinal chemistry practice. In parallel, virtual screening studies increasingly adopt unbiased target panels such as LIT‐PCBA to avoid artificial enrichment arising from decoy bias and to evaluate models under truly prospective‐like conditions [13]. Common molecular machine learning tasks and their recommended public benchmarks are summarized in Table 1.

**TABLE 1:** Drug discovery tasks and recommended benchmarking datasets (with key references).

Task	Example benchmarks/datasets	Notes	Key ref(s)
Property prediction	MoleculeNet; OGB‐MolHIV/MolPCBA	Use scaffold/time splits; report calibration and domain shift.	[11, 12]
Virtual screening	LIT‐PCBA	Prefer unbiased targets; avoid decoy bias; external structure checks.	[13]
De novo generation	GuacaMol; MOSES	Validity, uniqueness, novelty; multi‐objective goals; synthesizability.	[14, 15]
Conformation generation	GeoDiff tasks	RMSD and coverage; respect symmetry and chirality.	[16]
Text‐molecule tasks	MolT5 corpora	Captioning; text‐to‐molecule; leakage and alignment checks.	[17]

Abbereviations: AUROC, area under the receiver operating characteristic curve; PR‐AUC, area under the precision–recall curve. Table 1 summarizes common task families and widely used public benchmarks in drug discovery. For each benchmark, it is good practice to cite the dataset paper and to report the exact split protocol, especially for scaffold‐based and time‐aware splits.

## 3 What Counts as a Foundation or Multimodal Model in Chemistry?

Following the broader definition used in adjacent areas of machine learning, a foundation model in this domain is pretrained self‐supervised on large‐scale chemical and biological corpora and then adapted, via task heads, task conditioning, or lightweight adapters, to diverse downstream problems [1]. The key separation is between a computationally expensive, representation‐building pretraining stage and a comparatively inexpensive adaptation phase that specializes the model for property prediction, generation, screening, or protein‐related tasks.

In proteins, language models trained on massive sequence sets extract functional and structural signal directly from evolutionary variation. Models in the ESM family, trained solely on sequences, provide embeddings that support end‐to‐end structure prediction and supply features for clustering, annotation transfer, and generative design workflows [18, 19].

For small molecules, chemical language models extend these principles to SMILES/SELFIES. Masked‐token objectives, fragment‐aware masking, and data augmentation let the models internalize grammar‐level regularities and generalize across large, structurally diverse datasets [20]. After pretraining, the backbone is adapted for property prediction, docking‐score approximation, or controlled generation with simple conditioning layers, which is helpful when labeled data are limited.

**FIGURE 1:**
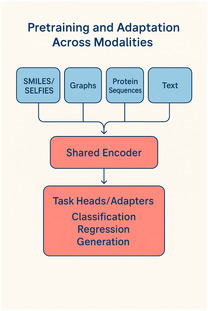
Pretraining and adaptation across modalities. Schematic overview of multimodal pretraining and downstream adaptation across modalities.

Text‐molecule models align natural‐language descriptions with chemical representations, enabling molecular captioning, zero‐shot retrieval, and text‐conditioned design from captions, assay summaries, or medicinal‐chemistry notes [17]. On the structural side, diffusion models formulated directly in 3D sample conformers or generate full molecules under E(3) symmetry, offering fine control over geometry and stereochemistry for conformer ensembles, scaffold elaboration, and pocket‐conditioned ligand design [16, 21]. A schematic overview of this multimodal pretraining and adaptation setup is shown in Figure 1. In practice, modality‐specific encoders map chemical strings, molecular graphs, protein sequences, and text into a shared representation space using masked modeling or contrastive alignment objectives. After pretraining, the same backbone can be adapted with lightweight heads or parameter‐efficient updates for classification, regression, retrieval, or generation. For example, protein language models in the ESM family provide embeddings that transfer to structure‐related tasks and target‐aware workflows [18, 19], while text‐molecule models such as MolT5 enable captioning and retrieval that support text‐conditioned exploration and prioritization [17]. MolT5, for instance, supports translation between molecules and natural language and enables text‐to‐molecule retrieval as well as captioning.

## 4 Practical Multimodality: Aligning Molecules, Proteins, and Text

Multimodal learning is useful when each modality captures distinct aspects of molecular behavior. In structure‐based design, this often means combining ligand representations, 2D graphs, or 3D coordinates, with protein information such as pocket geometry or sequence‐derived embeddings. Adding pocket context can improve ranking, pose awareness, and sensitivity to small chemical changes, provided that ligand‐protein alignment is learned without leakage. Protein language models such as ESM‐2 and structure surrogates like ESMFold enable inclusion of protein‐level signal even when experimental structures are unavailable, offering fast approximations of binding environments [18, 19].

Beyond ligand‐protein pairs, text represents an additional and increasingly valuable modality. Assay descriptions, high‐level protocols, compound series annotations, and medicinal‐chemistry notes contain implicit constraints that are difficult to encode in structural form alone. These sources provide hints about experimental conditions, target families, readout types, and outlier‐handling conventions, all of which can influence model predictions. The ability to integrate text therefore, allows models to condition on contextual cues that are orthogonal to geometric or sequence‐based signals.

Different modalities expose complementary strengths and limitations. Sequence‐only pretraining may miss stereochemistry, conformer preferences, and spatial motifs relevant for binding or reactivity [20], while 3D‐only approaches often suffer from sparse structural data, conformer uncertainty, and limited protein coverage. Multimodal schemes attempt to bridge these gaps: contrastive objectives associate molecules with captions, pocket embeddings, or assay‐level labels in a shared latent space; masked cross‐modal modeling enforces consistency between textual and structural descriptors; and cross‐attention between pocket and ligand streams captures interaction‐focused features without handcrafted terms. These strategies work best when alignment robustness is tested under distribution shift, ensuring that associations learned during pretraining remain stable as chemical series, assay types, or target classes change [17].

## 5 Generative Modeling and Diffusion in 3D

Deep generative chemistry has moved beyond SMILES autoregression. Score‐based and diffusion models now operate in 3D, denoising coordinates and atom types under E(3) symmetry while enforcing chemical validity by construction or by learned guidance [16, 21]. Such models are promising for conformation ensembles, scaffold elaboration near binding pockets, and pocket‐conditioned design, where geometry and stereochemistry matter. To be actionable, generative pipelines should report validity, uniqueness, and novelty alongside task‐oriented metrics and should verify synthesizability and stability with cheminformatics filters before expensive computation or synthesis [14, 15].

## 6 Evaluation Under Realistic Conditions

**FIGURE 2:**
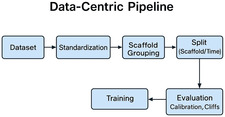
Data‐centric pipeline supporting split strategy and evaluation. Schematic overview of a data‐centric evaluation pipeline for molecular machine learning, highlighting standardization, deduplication, split strategies, and metric selection to reduce information leakage and support realistic benchmarking.

Reported gains often vanish outside random splits. Scaffold‐based and temporal splits better approximate medicinal‐chemistry practice by separating cores and respecting the arrow of time [11, 22]. Benchmarks such as MoleculeNet and OGB include such protocols, and task‐specific suites like GuacaMol, MOSES, and LIT‐PCBA help quantify generative quality and screening enrichment beyond superficial similarity [13, 14, 15]. Uncertainty and calibration deserve equal billing: conformal prediction yields valid per‐prediction confidence sets under mild assumptions [23], while modern classifiers are often miscalibrated unless explicitly corrected [24]. Activity cliffs, where small structural changes correspond to large potency shifts [25], should be audited, as average metrics can hide systematic blind spots. Target‐aware leakage (e.g., analog series spread across splits) should be checked with canonicalization, scaffold grouping, and deduplication pipelines. The overall data‐centric pipeline supporting these split strategies is summarized in Figure 2. Figure 2 summarizes a practical data‐handling pipeline that strongly influences whether evaluation is realistic. Standardization steps such as canonicalization, charge normalization, salt removal, and consistent stereochemistry are followed by deduplication and scaffold grouping to reduce analog‐series leakage across splits. The curated dataset can then be partitioned with random, scaffold‐based, or time‐aware protocols, and evaluated with metrics that capture ranking performance, calibration, and sensitivity to distribution shift. When available, time‐aware splits provide a simple proxy for prospective deployment conditions.

Here, common task families in molecular machine learning are organized by the splitting strategies, evaluation metrics, and public benchmarks most used in practice. For QSAR (quantitative structure–activity relationship) and ADMET, scaffold‐ or time‐aware splits are preferred because they limit structural leakage and capture temporal drift, while metrics such as AUROC, PR‐AUC, and calibration scores provide a fuller view of predictive reliability. Virtual‐screening tasks emphasize enrichment measures (EF1%, ROC enrichment) and typically rely on unbiased panels like LIT‐PCBA.

Generative‐modeling tasks require chemical validity checks alongside diversity metrics; validity, uniqueness, and novelty describe basic generator behavior, while conformal prediction or uncertainty measures flag unreliable regions of chemical space. Conformation‐generation tasks focus on geometric fidelity, often summarized by RMSD and coverage. Text‐conditioned tasks, captioning, and text‐to‐molecule retrieval use retrieval@k, BLEU, or BERTScore on corpora aligned across chemical and textual modalities.

Together, these entries provide an overview of how evaluation practices diverge across tasks and which benchmarks best represent each problem setting. In Table 2, EF1% denotes the enrichment factor at 1%, RMSD denotes root‐mean‐square deviation, and R@k denotes recall at k; BLEU and BERTScore are standard text generation metrics.

**TABLE 2:** Evaluation landscape for common drug discovery tasks (with representative benchmarks and key references).

Tasks	Recommended Splits / Metrics	Benchmarks	Key ref(s)
QSAR	Scaffold/Time‐aware; AUROC · PR‐AUC; Calibration	MoleculeNet; OGB‐MolHIV/MolPCBA	[11, 12]
ADMET	Scaffold/Time‐aware; AUROC; Calibration	MoleculeNet	[11]
Virtual screening	EF1% · ROC enrichment; Calibration error	LIT‐PCBA	[13]
Molecule generation	Conformal set size; Validity · Uniqueness · Novelty	GuacaMol; MOSES	[14, 15]
Conformation generation	RMSD; Coverage	GeoDiff tasks	[16]
Text‐to‐molecule	Caption split; Retrieval (R@k); BLEU/BERTScore	MolT5 corpora	[17]

## 7 Data Standards, Provenance, and Reproducibility

For molecular foundation and multimodal models, performance is only as reliable as the data pipelines behind them. Standardized representations, explicit provenance, and transparent curation are therefore as important as architecture. The FAIR principles, Findable, Accessible, Interoperable, Reusable, provide a practical guide: datasets should include machine‐readable metadata, stable identifiers, and clear licenses so that others can reconstruct splits and reproduce results [26]. This requires reporting exact database releases, the cheminformatics toolkit, and settings used for standardization, and any filtering rules applied.

Provenance is crucial when combining heterogeneous sources, public databases, internal experiments, and literature‐derived assays into large pretraining corpora. Without tracking origin, assay format, and processing history, it becomes difficult to explain differences in model behavior across targets or chemical series. Efforts such as SURF and the Open Reaction Database show how structured metadata make experimental records both human‐ and machine‐readable [27, 28]. Similar standards for bioactivity and structural datasets would likewise improve traceability.

Reproducibility also depends on how data are split and evaluated. Changes in scaffold definitions, time windows, or leakage checks can shift benchmark difficulty, yet are often underreported. Fair comparisons require specifying standardization and deduplication, treatment of analog series, the splitting strategy, and random seeds. Ideally, scripts that rebuild the dataset, from raw sources to final partitions, should accompany model code. As models and datasets scale, strict bitwise reproducibility becomes less important than auditability: independent groups should recover similar trends and identify what drives improvements. This relies on clear documentation of pretraining objectives, parameter counts, adapter strategies, and compute budgets. Table 3 offers a concise reporting checklist covering splitting, provenance, and uncertainty. Reporting the data source and exact release, preprocessing settings, split strategy, and uncertainty or calibration methods is often sufficient to prevent common reproducibility failures.

**TABLE 3:** Reporting checklist for molecular machine learning studies.

Item	What to report
Data provenance	Source, license, exact release (e.g., ChEMBL vXX), curation steps (RDKit standardization, salt removal), exclusions.
Splitting protocol	Random vs. scaffold vs. temporal; rationale; seeds; leak checks; duplicates removed.
Model and pretraining	Architecture; tokenizer/featurization; unsupervised objective; parameter counts; adapter strategy; compute.
Evaluation and uncertainty	Primary metrics; calibration; conformal prediction; activity‐cliff analysis; class imbalance handling.
Reproducibility	Code, data snapshots, scripts; environment; ablations; external test or prospective evaluation.

*Note:* The key reporting items are summarized in Table 3 to support auditable comparisons across datasets, splits, and model variants. In practice, reporting the data source and release, preprocessing and standardization settings, deduplication criteria, and the exact split script is often sufficient to make results reproducible. When possible, uncertainty or calibration methods and a brief compute budget description should also be included.

## 8 Summary

This mini‐review examined how recent foundation and multimodal approaches are reshaping molecular informatics across representation, modeling, and evaluation. String and graph encodings, curated repositories such as ChEMBL, and standardized cheminformatics pipelines provide scalable inputs, while geometry‐aware and equivariant architectures capture stereochemistry, conformational preferences, and local spatial constraints. Large‐scale pretraining on chemical and biological corpora supports transfer to QSAR/ADMET, virtual screening, reactivity prediction, and generative design, and protein language models now supply sequence‐derived structure surrogates that integrate naturally with ligand features in structure‐based workflows. Multimodal learning aligns molecules with protein pockets, evolutionary context, and textual descriptors from assays or medicinal chemistry, improving pose awareness, retrieval, and captioning. Diffusion models extend generation to 3D, enabling conformer ensembles, scaffold elaboration, and pocket‐conditioned exploration of chemical space. Evaluation practices, scaffold and time splits, calibration, conformal prediction, activity‐cliff inspection, and unbiased benchmarks, remain central to determining whether improvements persist under distribution shift. Finally, data standards, transparent curation, and clear documentation of preprocessing and provenance are essential for reproducibility and for deploying these models across heterogeneous tasks and laboratories.

## 9 Outlook

Multimodal foundation training is becoming practical across chemical strings, molecular graphs, 3D conformers, protein sequences, pocket representations, and text. Future progress will depend increasingly on data governance and evaluation realism, rather than on architecture alone.

Benchmarks should move beyond average scores by requiring leakage‐aware splits, calibration reporting, and diagnostics that expose failure modes such as activity cliffs and target‐aware leakage. Multimodal settings add further challenges, because cross‐modal duplication can silently inflate retrieval and captioning results.

As models and datasets scale, attention should also shift to dataset provenance, licensing, and reproducibility. Transparent reporting, consistent preprocessing, and shareable split scripts are essential for translating benchmark improvements into prospective performance in drug discovery.

## Conflicts of Interest

The authors declare no conflicts of interest.

## Data Availability

Data sharing not applicable to this article as no datasets were generated or analysed during the current study.
